# Ecological pathways to prevention: How does the SASA! community mobilisation model work to prevent physical intimate partner violence against women?

**DOI:** 10.1186/s12889-016-3018-9

**Published:** 2016-04-16

**Authors:** Tanya Abramsky, Karen M. Devries, Lori Michau, Janet Nakuti, Tina Musuya, Ligia Kiss, Nambusi Kyegombe, Charlotte Watts

**Affiliations:** Gender Violence and Health Centre, London School of Hygiene and Tropical Medicine, 15-17 Tavistock Place, London, WC1H 9SH UK; Raising Voices, 16 Tufnell Drive, Kamwokya, P.O Box 6770 Kampala, Uganda; Centre for Domestic Violence Prevention, 16 Tufnell Drive, Kamwokya, P.O Box 6770 Kampala, Uganda

**Keywords:** Violence prevention, Impact evaluation, Community mobilisation, Intimate partner violence, Uganda, Pathways analysis, Gender based violence, East Africa

## Abstract

**Background:**

Intimate partner violence (IPV) against women is a global public health concern. While community-level gender norms and attitudes to IPV are recognised drivers of IPV risk, there is little evidence on how interventions might tackle these drivers to prevent IPV at the community-level. This secondary analysis of data from the SASA! study explores the pathways through which SASA!, a community mobilisation intervention to prevent violence against women, achieved community-wide reductions in physical IPV.

**Methods:**

From 2007 to 2012 a cluster randomised controlled trial (CRT) was conducted in eight communities in Kampala, Uganda. Cross-sectional surveys of a random sample of community members, aged 18–49, were undertaken at baseline (*n* = 1583) and 4 years post intervention implementation (*n* = 2532). We used cluster-level intention to treat analysis to estimate SASA!’s community-level impact on women’s past year experience of physical IPV and men’s past year perpetration of IPV. The mediating roles of community-, relationship- and individual-level factors in intervention effect on past year physical IPV experience (women)/perpetration (men) were explored using modified Poisson regression models.

**Results:**

SASA! was associated with reductions in women’s past year experience of physical IPV (0.48, 95 % CI 0.16–1.39), as well as men’s perpetration of IPV (0.39, 95 % CI 0.20–0.73). Community-level normative attitudes were the most important mediators of intervention impact on physical IPV risk, with norms around the acceptability of IPV explaining 70 % of the intervention effect on women’s experience of IPV and 95 % of the effect on men’s perpetration. The strongest relationship-level mediators were men’s reduced suspicion of partner infidelity (explaining 22 % of effect on men’s perpetration), and improved communication around sex (explaining 16 % of effect on women’s experience). Reduced acceptability of IPV among men was the most important individual-level mediator (explaining 42 % of effect on men’s perpetration).

**Conclusions:**

These results highlight the important role of community-level norm-change in achieving community-wide reductions in IPV risk. They lend strong support for the more widespread adoption of community-level approaches to preventing violence.

**Trial registration:**

ClinicalTrials.gov, NCT00790959. Registered 13th November 2008.

The study protocol is available at: http://www.trialsjournal.com/content/13/1/96

**Electronic supplementary material:**

The online version of this article (doi:10.1186/s12889-016-3018-9) contains supplementary material, which is available to authorized users.

## Background

Violence against women is widely recognised as a serious human rights and public health concern, one that is associated with a range of poor health outcomes including HIV infection [1–4]. Intimate partner violence (IPV) is the most common form of violence against women, with recent estimates suggesting that 30 % of ever partnered women worldwide will experience physical and/or sexual violence by an intimate partner during their lifetime [5].

Ecological frameworks are now extensively used to describe the multiple levels (societal, community, relationship, individual) at which factors operate to influence IPV risk [6, 7], and there is growing recognition that as well as targeting individuals, violence prevention strategies must address the social, cultural and economic contexts in which IPV occurs. Community- and societal-level factors shown empirically to be linked to women’s risk of experiencing or men’s risk of perpetrating IPV include norms relating to the acceptability of wife beating [8–12] and male authority over female behaviour [9], norms granting men economic and decision-making power in the household [13], low levels of autonomy among women [14], lack of easy access to divorce for women [13], low literacy rates [15], low levels of female education [9, 11], high levels of poverty and unemployment [16], and lack of community sanctions against IPV [17]. These contexts in turn engender many of the individual- and relationship-level factors associated with increased risk of IPV, such as childhood experience of abuse or exposure to violence between parents, attitudes accepting of violence against women, low levels of education, harmful use of alcohol or drugs, economic stress, conflict or dissatisfaction in a relationship, male dominance in the family, and men having multiple partners [7, 18–20]. And yet, to date, there is little evidence on how interventions might engage with community-level drivers of IPV-risk in order to achieve community-wide reductions in violence.

Extant IPV prevention interventions which have sought to challenge regressive gender norms or address women’s economic dependence on men, have often comprised small group based workshops, sometimes coupled with livelihood strategies. They have for the most part targeted enrolled individuals, and - while they have met with success in changing *attitudes*, relationship dynamics and behaviours among group attendees – their impacts on *norms* within the wider community have not been evaluated [21–24].

Community-level approaches commonly used to try and change norms include awareness raising campaigns, such as the annual 16 Days of Activism against Gender Violence, which though helpful in bringing discussions of violence out into the open and providing a focus for local action, rarely have sufficient intensity or theoretical grounding to transform norms or change behaviours [7]. ‘Edutainment’ programmes such as Soul City in South Africa and Sexto Sentido in Nicaragua [25, 26] - that use media and/or entertainment to reinforce social change messages at a community level - are also an increasingly popular approach to changing norms and behaviours. Such programmes arguably offer the greatest potential for change when used as part of broader community mobilisation strategies, which use diverse strategies to reach and engage as many people as possible [21]. Because the design, implementation and evaluation of such interventions are so complex, however, there is scant evidence on their potential effectiveness and the mechanisms through which they might work [21].

We recently reported the results of the SASA! Study, a cluster randomised controlled trial to assess the community level impacts of SASA!, a community mobilisation intervention to prevent violence against women and reduce HIV-risk behaviours in Kampala, Uganda [27]. After just under three years of intervention programming, women in intervention communities were 52 % less likely to report past year experience of physical IPV, compared to women in control communities (adjusted risk ratio 0.48, 95 % CI 0.16-1.39), and also somewhat less likely to report past year experience of sexual IPV (aRR 0.76, 0.33–1.72). Though an increase in inter-community variation in prevalence of these outcomes over the course of the study reduced study power to obtain statistically significant results in relation to IPV, large effect sizes and consistency in direction of effect between these, other primary outcomes and secondary violence outcomes are strongly suggestive of an intervention effect on levels of IPV. The study also found statistically significant impacts on male and female attitudes towards the acceptability of IPV, and reductions in past year sexual concurrency among men [27]. This was the first trial in sub-Saharan Africa, shortly followed by a second study from Uganda [28], to show community-level impacts on IPV-related attitudes and behaviours, with the effects not confined to individuals reporting direct exposure to the intervention - effect sizes were similar whether the analysis was restricted to explore effect among individuals reporting above a threshold level of exposure to the intervention, or done on an Intention to Treat (ITT) basis whereby all members of intervention communities (both exposed and unexposed) were included [27].

Having demonstrated that community level change is possible*,* this paper explores the potential pathways - changes in individual-, relationship- and community-level attitudes and behaviours - through which reductions in IPV were achieved in the SASA! study communities.

## Methods

### Study setting

The SASA! Study was conducted between November 2007 and May 2012 in the Rubaga and Makindye Divisions of Kampala, Uganda. Uganda has a high prevalence of IPV and HIV/AIDS, and patriarchal norms are a dominant aspect of the sociocultural environment. In Kampala, 9.5 % of women aged 15–49 are estimated to be living with HIV [29], and 45 % of ever-married women aged 15–49 report having experienced physical and/or sexual violence by an intimate partner at some point in their lives [30].

### The SASA! intervention

The *SASA! Activist Kit for Preventing Violence against Women and HIV* [31] is a community mobilisation intervention seeking to change community attitudes, norms and behaviours that result in gender inequality, violence and increased HIV vulnerability for women. A central focus of the intervention is to promote a critical analysis and discussion of power and power inequalities - not only the ways in which men and women may misuse power and the consequences of this for their relationships and communities, but also how people can use their power positively, to affect and sustain change at an individual and community level. SASA! was designed by Raising Voices (http://raisingvoices.org/) and implemented in Kampala by the Centre for Domestic Violence Prevention (CEDOVIP) (http://raisingvoices.org/activism/local/).

**Fig. 1:**
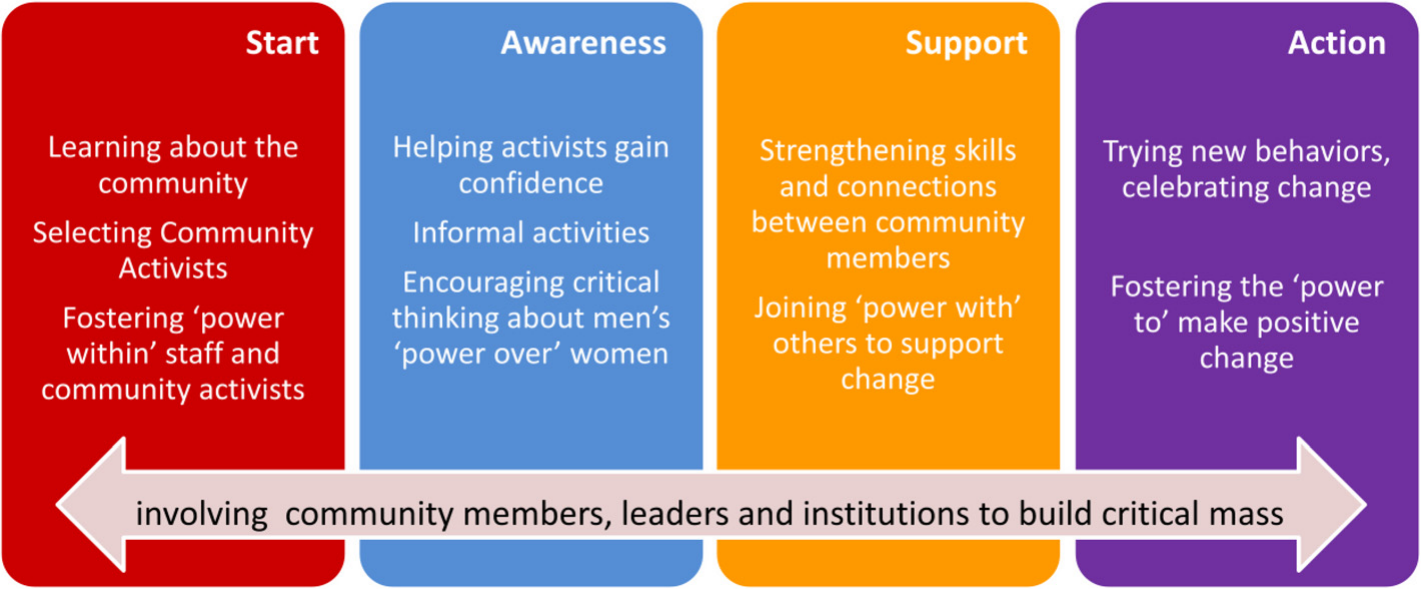
4 phases of SASA!

Designed around the Ecological Model of Violence [7], SASA! recognises that IPV results from the complex interplay of factors operating at the individual, relationship, community and societal levels. The approach therefore supports whole communities through a phased process of change, systematically involving a broad range of stakeholders. SASA!, meaning ‘Now’ in Kiswahili, is an acronym for the four phases of the approach - Start, Awareness, Support, Action (see Fig. 1) - with the phases analogous to the processes set out by Prochaska et al. (1992) in their individual-level behaviour-change Stages of Change Theory [32].

Throughout this process SASA! intervention staff work with four groups of actors: community activists (CAs) selected from among the more progressive men and women rooted in the community, who work voluntarily to facilitate and promote SASA! activities; community leaders including *ssengas* (traditional marriage counsellors) who, as religious, cultural, governmental and other types of local leaders, are encouraged to integrate ideas of gender and power into their leadership roles; professionals such as health care providers and police officers, who provide direct prevention and response services; and institutional leaders who have the power to implement policy changes within their institutions.

SASA! entails the selection, ongoing training, and mentoring of these individuals and groups, to help improve their knowledge, communication skills, and motivation to participate in mobilising their communities to address gender inequality and violence. They are introduced to, and then supported to introduce into their communities, new concepts of power, encouraging analysis of gender-related imbalances of power through four strategies: Local Activism, Media and Advocacy, Communication Materials, and Training. As part of this, CAs and leaders are supported by CEDOVIP staff to conduct a range of local activism activities, including (but not limited to) public events such as community dramas, discussions and meetings; small group activities; one-on-one ‘quick chats’; door-to-door discussions; trainings; poster discussions; and film and soap opera shows. Similarly, the police, health workers and other professionals receive training and are supported in efforts to improve the provision of services. In this way, community members are exposed to SASA! ideas repeatedly and in diverse ways within the course of their daily lives, from people they know and trust as well as from more formal sources within their communities. The specifics of intervention activities develop and continually evolve in response to community priorities, needs and characteristics.

In the context of this CRT, eight CAs (four male and four female) were recruited in each intervention parish, and trained and supported to deliver the intervention. For sampling purposes (see below for detail) an identical recruitment process was used in control sites, but selected individuals (passive volunteers) went on to receive just one session of basic health education or children’s rights training every three months. Programming continued for the duration of the study (equating to approximately 2.8 years of programming after taking into account interruptions caused by political disturbances). Monitoring data show that over the course of the study SASA! activists led more than 11,000 activities, and reached an estimated 260,000 community members (unpublished process and monitoring data).

### Trial design

The cluster randomised trial ran from 2007 to 2012. The design is described in detail elsewhere [33]. Briefly, eight sites (consisting of one or two administrative parishes) were pair matched on the basis of estimated population density and stability/mobility of the population. One from each pair was randomised to receive the intervention, and the other designated as a control.

Two cross-sectional surveys of community members were carried out in all sites, one prior to intervention implementation (baseline) and one approximately four years later (follow-up). The sampling frame for the surveys comprised households situated in the enumeration areas (EA) in which community activists (or ‘passive volunteers’) lived. For the baseline survey a stratified random sample of 8 community activist EAs per site was selected (64 in total; 32 per intervention arm); at follow-up, due to increased funding we were able to sample all EAs in which CAs were recruited at the start of the study (96 in total; 48 per intervention arm). 35 households per EA were then randomly selected, and a maximum of one eligible person per household selected to complete the survey. In the interest of respondent safety, to reduce the chance that men in the immediate locality were aware of the nature of the questions in the survey and the potential disclosures that women may make, an exclusively female sample was drawn from EAs in which female community activists lived, and an exclusively male sample drawn around male community activists. Data do not therefore pertain to men and women in the same relationships, nor living in the same immediate localities.

One thousand, five hundred eighty-three respondents were interviewed at baseline and 2,532 at follow-up. Intervention effects on primary outcomes (including women’s past year experiences of physical and sexual IPV) were assessed using an adjusted cluster-level intention to treat (ITT) analysis which compared outcomes in intervention and control communities at follow-up [27].

The study received ethical approval from institutional review boards at the London School of Hygiene and Tropical Medicine (UK) (ref.5210), Makerere University (Uganda) (ref. 2007–101) and the Uganda National Council for Science and Technology (SS 2048). Approval to work in the study communities was obtained from local government offices and leaders, and individual-level written consent was obtained prior to each interview.

### Hypothesized pathways to reduced IPV

**Fig. 2:**
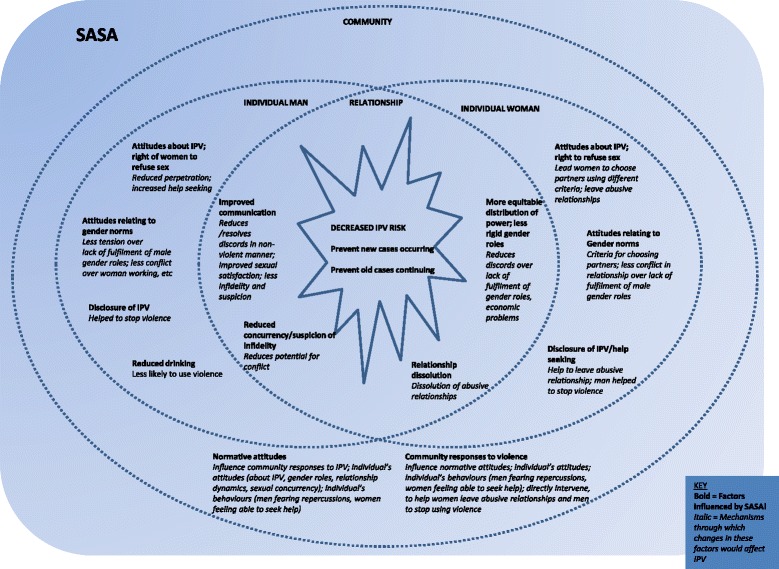
Conceptual framework – pathways to reduced IPV risk

The conceptual framework for this analysis (Fig. 2) draws on ecological models of risk factors for IPV, the SASA! Study logic model which laid out a theoretical framework for how SASA! would achieve its long term intended outcomes (Additional file 1) [33], and qualitative research on pathways of change conducted with SASA! community members [34]. Key measurable intermediate outcomes are laid out in an ecological framework, with both primary and secondary prevention of IPV as the central goals. The potential mediators (Table 1) comprise community-level factors (responses to IPV occurring in the community, and normative attitudes towards IPV, women’s right to refuse sex or request condom use, and broader gender roles); relationship-level factors (communication between partners, relationship power dynamics, extra-spousal sex partners, relationship dissolution); and individual-level factors for both men and women (drinking behaviour and attitudes relating to IPV, a woman’s right to refuse sex and broader gender roles).

**Table 1:** Measures of IPV and potential community-, relationship- and individual-level mediators of intervention effect on IPV

Concept being measured	Indicator	Items in composite index
IPV OUTCOMES (among women/men who have had a regular/casual partner in the past year)		
Women’s experience of IPV	Women’s past year experience of physical IPV	Reports that her partner/most recent partner has done at least one of the following things to her in the past year: • Slapped her or thrown something at her that could hurt her • Pushed her or shoved her or pulled her hair • Hit her with his fist or something else that could hurt her • Kicked her, dragged her or beat her up • Choked or burnt her on purpose • Threatened to use or actually used a gun, knife or other weapon against her • Threatened to use or actually used a panga (stick) against her
Men’s perpetration of IPV	Men’s past year perpetration of IPV	Fills in anonymous card that ‘yes’ he has ‘used violence on your (partner) [most recent partner] in the last 12 months (last 12 months of your most recent relationship).’ ^a^
MEDIATORS		
Community-level mediators (EA-level^**^ aggregate prevalence)		
Community responses to prevent violence	Okay for others in community to intervene if they know IPV is occurring	Among all respondents in EA, percentage who answers ‘yes’ to the question: ‘If a husband beats up his wife, should others outside the couple intervene?’
	People who have witnessed/heard violence who have responded appropriately (among those who have seen or heard IPV in their community)	Among respondents in EA who had seen or heard IPV happening in their community, percentage who reported that: • ‘Yes’ they ‘did something to try to help’ AND reported doing so with at least one of the following responses: • Gathered other people in the community to help • Knocked on the door to stop/distract the couple from fighting • Separated the couple that was fighting • Informed a community activist, ssenga, LC or police or any other authority • Talked to the woman afterwards and told her to talk to a family member, friend, community activist, LC or ssenga or any other authority • Talked to the woman afterwards and asked her how she wanted to be helped • Talked to the man afterwards and told him that violence is never acceptable • Talked to the man afterwards and told him to talk to a family member, friend, community activist, LC or ssenga or any other authority • Talked to the man afterwards and tried to help him stop using violence
Norms around violence	Acceptable for a man to use violence against his partner	Among all respondents in EA, percentage who answer that ‘yes’, a man has good reason to hit his wife in at least one of the following scenarios: • She disobeys him • She answers back to him • She disrespects his relatives • He suspects that she is unfaithful • He finds out she has been unfaithful • She spends time gossiping with neighbours • She neglects taking care of the children • She doesn’t complete her household work to his satisfaction • She refuses to have sex with him • She accuses him of infidelity • She tells his secrets to others in the community • He is angry with her
Norms around women’s control over sex	Acceptable for a woman to refuse sex with her partner	Among all respondents in EA, percentage who answer that ‘yes’ in their opinion it is acceptable if a married woman refuses to have sex with her husband if she doesn’t feel like it.
	Okay for a woman to ask her husband to use a condom	Among all respondents in EA, percentage who answer that ‘yes’ it is acceptable for a married woman to ask her husband to use a condom.
Broader gender norms	Others in community would respect a man who made decisions jointly with his wife	Among all respondents in EA, percentage who answer that ‘yes’, if a husband told his friends that he makes decisions jointly with his wife, his friends would respect him.
	Man’s role to decide if his wife can work	Among all respondents in EA, percentage who answer that ‘yes’ they think it is a husband’s role to decide whether or not his wife can work outside the home.
Relationship level mediators (for those partnered in past year)		
Communication	Discuss things that happen in day	Answers ‘yes’ in the last 12 months they and their partner discuss things that happen to the respondent during the day, AND things that happen to their partner during the day.
	Discuss worries	Answers ‘yes’ in the last 12 months they and their partner discuss the respondent’s worried or feelings.
	Discuss what you both like during sex	Answers ‘yes’ that over the last 12 months they have openly asked their partner about what he/she likes during sex, AND that they have openly told their partner about what they themselves like during sex.
	Appreciate work partner does around house (where applicable)	Answers ‘many times’ (versus ‘none’ or ‘a few’) to the question of how many times they have shown appreciation for the work their partner does inside the home.
	Appreciate work partner does outside house (where applicable)	Answers ‘many times’ (versus ‘none’ or ‘a few’) to the question of how many times they have shown appreciation for the work their partner does outside the home.
Power dynamics	Joint decision making	Answers that ‘most of the time’ or ‘all of the time’ in the last 12 months they have made decisions jointly with their partner on important issues, such as where they stay/live or what school the children attend
	Man helps around house (among cohabiting couples)	Answers that ‘yes’ in the last 12 months the male partner/male respondent has regularly helped with any of the housework.
	Woman refused a job because husband doesn’t want her to work	Answer that ‘yes’ in the last 12 months the female respondent/female partner has given up or refused a job for money outside the home because her partner did not want her to work.
	Woman participated in deciding how household finances spent (among cohabiting couples)	Female respondent answers that ‘yes’ in the last 12 months she has participated in deciding how the family finances were spent. Male respondent answers that ‘no’ in the last 12 months he hasn’t made all decisions regarding how the family finances were spent independent of his wife.
Additional sex partners	Concurrent partners (among non-polygamous partnered respondents)	Answers that ‘yes’ they have had a sexual relationship with any other person in the last 12 months while being with their partner.
	Male partner often suspicious that female partner is unfaithful	Reports that ‘yes’ in the last 12 months the male partner/male respondent has often been suspicious that the female respondent/female partner is unfaithful.
Relationship dissolution	Separated/divorced in past year (among those who have been married, lived together with someone as if married, or had a regular partner at some point in the last 12 months)	Among those who have been married or in a relationship at some point in the past year, those who report being currently ‘separated’, ‘divorced’ or ‘single’.
Individuals (for all respondents, separately for men and women)		
Attitudes around violence	Acceptable for a man to use violence against his partner	Answers ‘yes’, a man has good reason to hit his wife in at least one of the following scenarios: • She disobeys him • She answers back to him • She disrespects his relatives • He suspects that she is unfaithful • He finds out she has been unfaithful • She spends time gossiping with neighbours • She neglects taking care of the children • She doesn’t complete her household work to his satisfaction • She refuses to have sex with him • She accuses him of infidelity • She tells his secrets to others in the community • He is angry with her
	Okay for a woman to tell others if she is experiencing violence	Answers that ‘yes’, if a married woman has been beaten up by her husband, it is okay for her to tell others.
Attitudes towards women’s control over sex	Acceptable for a woman to refuse sex with her partner	Answers that ‘yes’ in their opinion it is acceptable if a married woman refuses to have sex with her husband if she doesn’t feel like it.
	Okay for a woman to ask her husband to use a condom	Answers that ‘yes’ it is acceptable for a married woman to ask her husband to use a condom.
Broader gender attitudes	Others in community would respect a man who made decisions jointly with his wife	Answers that ‘yes’, if a husband told his friends that he makes decisions jointly with his wife, his friends would respect him.
	Man’s role to decide if his wife can work	Answers that ‘yes’ they think it is a husband’s role to decide whether or not his wife can work outside the home.
Behaviours	Drunk at least once a month	Answers that in the last 12 months they have been drunk ‘most days’, ‘weekly’ or ‘once a month’ (versus ‘never’ or ‘less than once a month’)
	Woman experiencing (man perpetrating) violence who has told someone (among those experiencing/perpetrating IPV)	Among respondents who report IPV experience (women)/perpetration (men), those who report that ‘yes’ they have told someone about any of their experiences.

^a^ Male disclosure of IPV perpetration in the main questionnaire was extremely low, especially in intervention communities. Therefore, for the purpose of this analysis, when looking at pathways to reduced male perpetration, we have instead used violence data from cards that respondents were asked to fill out anonymously and place in a sealed envelope at the end of the interview

**EA = census enumeration area

Pathways of effect were conceptualised using an ecological framework for the principal reason that SASA! is a community level intervention, and potential pathways of effect may operate at the community, relationship and individual-levels. For example, a woman’s IPV risk could decrease because changes in her own attitudes lead her to choose a non-violent partner. However, her IPV risk might also decrease without any antecedent changes in her own attitudes or behaviours, if for example her partner attends intervention activities and becomes less likely to perpetrate IPV against her, or if community change leads others in the community to intervene when they become aware she has experienced IPV. Furthermore, the absence of arrows in ecological models illustrates the interplay of factors operating at different ecological levels – for example, community norm change may affect individuals’ behaviours within relationships, which in turn feed back into community norm change.

With a complex community-level intervention such as SASA!, pathways of effect are also likely to vary from person to person, depending on their characteristics, experiences of the intervention, and current situations. For example, pathways that lead to cessation of IPV among women already experiencing violence in their relationship will likely differ markedly from pathways that reduce risk of onset of new IPV among women not previously experiencing violence. Furthermore, multiple pathways of change may operate concurrently within any one individual. For this reason, this paper does not attempt to ‘model’ a pathway by laying out a sequential chain of causation or proportioning out causality among the various factors. Instead, we attempt to outline which mediating factors played a role in reducing IPV.

### Outcome and mediator measures

Table 2 presents the survey questions used to construct the IPV outcomes. Briefly, women’s past year experience of IPV was measured using questions on experiences of specific violent acts by a partner (based on instruments used in the WHO Multi-country Study on Women’s Health and Domestic Violence and the Uganda Demographic and Health Survey (DHS)) [35, 36]. Since baseline data from the SASA! study suggested that men are likely to underreport perpetration of such acts [37], and indeed male disclosure of IPV was extremely low in the follow-up survey especially in intervention communities, we used an alternative method to measure men’s past year perpetration of IPV. We instead used violence data from cards that respondents were asked to fill out anonymously and place in a sealed envelope at the end of the interview. Details of the questions used to measure potential mediator variables are also presented in Table 2.

**Table 2:** Estimates of effect on IPV, comparing outcome in intervention versus control communities

	Baseline		Follow-up		
IPV outcome indicators	Intervention	Control	Intervention	Control	aRR^a^ (95 % CI)
	*n/N (%)*	*n/N (%)*	*n/N (%)*	*n/N (%)*	
Women’s past year experience of physical IPV (among those partnered in past year)	75/302 (25 %)	57/273 (21 %)	46/504 (9 %)	93/424 (22 %)	0.48 (0.16 to 1.39)
Men’s past year perpetration of IPV (among those partnered in past year)	95/309 (31 %)	101/326 (31 %)	123/617 (20 %)	256/523 (49 %)	0.39 (0.20–0.73)

^a^Risk ratios calculated at the cluster-level, adjusted for community-pair, and weighted according to the number of observations per village. Adjusted risk ratios generated on the basis of expected number of events from a logistic regression model on individual data with independent variables including age, marital status and EA-level summary baseline measure of IPV

### Statistical analysis

Intervention impacts on women’s past year experience and men’s past year perpetration of physical IPV were estimated using an adjusted cluster-level ITT analysis controlling for site pair, age, marital status and baseline EA-level prevalence of the outcome (as per the primary trial analysis) [27]. We explored the role of potential mediators using a three stage approach. We first used the same adjusted cluster-level ITT analysis method to estimate intervention impact on the intermediate outcomes identified in our conceptual framework. The analysis was performed separately for women and men. Individual- and relationship-level outcomes were binary, with adjusted risk ratios used as the measure of intervention impact. Community-level outcomes were continuous measures (enumeration area-level prevalence of each outcome), with intervention impact thus estimated using adjusted mean differences.

We then explored the associations between each of these intermediate outcomes and women’s past year experience of physical IPV (for variables reported by women)/men’s past year perpetration of IPV (for variables reported by men). Risk ratios were estimated using individual-level modified Poisson regression models adjusted for site-pair, intervention arm, age, marital status, education, and childhood experiences/witnessing of abuse. Sandwich variance estimators were used to both account for intra-cluster correlation at the enumeration-area level and correct for variance overestimation that occurs when Poisson regression is applied to binary data [38]. Interactions between intervention arm and the risk factor of interest were also checked to assess if risk factors were related to IPV similarly in both intervention and control communities. As no interactions were found, overall results for all communities are reported.

Finally, we modelled the effect of SASA! on past year physical IPV (experience for women/perpetration for men), adjusting for each potential pathway variable separately. We examined the extent to which each variable’s inclusion in the basic model (including site-pair, age, marital status and EA-level baseline prevalence of IPV) attenuated intervention impact on IPV - interpreting greater attenuation as suggestive of the increased importance of that variable as a mediator of intervention impact on IPV. In order to allow more flexibility to explore pathways, modified Poisson regression (as described above) was used, in place of a cluster-level analysis.

All analyses were performed using Stata 13.

## Results

Response rates for both the baseline and follow-up surveys were high (Additional file 2). At follow-up, 600 women and 768 men were successfully interviewed in intervention communities (99 %), and 530 women and 634 men in control communities (98 %). At both time-points there were high levels of comparability between intervention and control communities with respect to socio-demographic data (see Additional file 3 for descriptive data on the study sites and survey respondents). Prior to intervention implementation, intervention and control communities were also similar with respect to levels of IPV and the mediators for which baseline data were available (see Tables 2, 3 and 4) (although women in intervention communities were slightly more likely to report participating in deciding how household finances were spent).

**Table 3:** Estimates of intervention effect on potential mediators among women, comparing outcome in intervention versus control communities

	Baseline^a^		Follow-up		
	Intervention	Control	Intervention	Control	aRR^b^ (95 % CI)
COMMUNITY LEVEL^c^	*EA-level mean risk % (sd) n = 16*	*EA-level mean risk % (sd) n = 16*	*EA-level mean risk % (sd) n = 23*	*EA-level mean risk % (sd) n = 24*	*Mean difference (95 % CI)*
*Community responses to prevent violence:*					
Okay for others in community to intervene if they know IPV is occurring	-	-	79.2 (7.1)	58.7 (6.5)	20.3 (10.2–30.4)
People who have witnessed/heard violence who have responded appropriately	-	-	47.2 (16.3)	29.8 (13.6)	13.0 (−14.6–40.6)
*Norms around violence:*					
Acceptable for a man to use violence against his partner	57.0 (15.3)	59.1 (15.5)	28.1 (6.7)	51.1 (12.6)	−26.7 (−49.6– −3.7)
*Norms around women’s control over sex:*					
Acceptable for a woman to refuse sex with her partner	40.4 (14.7)	35.3 (15.1)	91.3 (3.2)	74.7 (10.3)	18.4 (6.0–30.9)
Okay for a woman to ask her husband to use a condom	-	-	78.5 (3.2)	59.2 (7.3)	20.4 (13.5–27.4)
*Broader gender norms:*					
Others in community would respect a man who made decisions jointly with his wife	-	-	75.2 (0.5)	57.2 (16.6)	22.8 (−2.7–48.3)
Man’s role to decide if his wife can work	-	-	39.3 (2.3)	58.6 (9.9)	−21.9 (−36.9– −7.0)
RELATIONSHIP LEVEL (PARTNERED IN PAST YEAR)	*n/N (%)*	*n/N (%)*	*n/N (%)*	*n/N (%)*	*aRR (95 % CI)*
*Communication:*					
Discuss things that happen in day	243/605 (80 %)	232/274 (85 %)	402/482 (83 %)	269/398 (68 %)	1.23 (1.01–1.48)
Discuss worries	255/305 (84 %)	231/274 (84 %)	433/482 (90 %)	295/398 (74 %)	1.21 (1.02–1.44)
Discuss what you both like during sex	-	-	321/481 (67 %)	183/398 (46 %)	1.49 (0.91–2.43)
Appreciate work partner does around house	-	-	269 /397 (68 %)	155/303 (51 %)	1.27 (1.08–1.50)
Appreciate work partner does outside house	-	-	346/410 (86 %)	244/308 (79 %)	1.08 (0.97–1.19)
*Power dynamics:*					
Joint decision making	219/266 (82 %)	205/246 (83 %)	279/421 (66 %)	154/332 (46 %)	1.42 (1.14–1.76)
Man helps around house	156/292 (53 %)	159/272 (58 %)	285/392 (73 %)	164/299 (55 %)	1.33 (0.94–1.88)
Woman refused a job because husband doesn’t want her to work	76/300 (25 %)	52/273 (19 %)	54/454 (12 %)	65/376 (17 %)	0.78 (0.15–4.10)
Woman participated in deciding how household finances spent	216/297 (73 %)^*^	169/272 (62 %)^*^	321/406 (79 %)	217/313 (69 %)	1.12 (1.01–1.24)
*Additional sex partners:*					
Concurrent partners	18/247 (7 %)	8/215 (4 %)	25/429 (6 %)	20/341 (6 %)	1.25 (0.37–4.22)
Male partner often suspicious that female partner is unfaithful	-	-	68/504 (13 %)	98/425 (23 %)	0.65 (0.24–1.73)
*Relationship dissolution:*					
Separated/divorced in past year	8/299 (3 %)	3/264 (1 %)	9/486 (2 %)	17/401 (4 %)	0.44 (0.08–2.52)
INDIVIDUALS (PARTNERED IN PAST YEAR)					
*Attitudes around violence:*					
Acceptable for a man to use violence against his partner	181/304 (60 %)	166/274 (61 %)	168/504 (33 %)	260/426 (61 %)	0.56 (0.38–0.82)
Okay for a woman to tell others if she is experiencing violence	-	-	409/504 (81 %)	241/427 (56 %)	1.45 (1.22–1.72)
*Attitudes towards women’s control over sex:*					
Acceptable for a woman to refuse sex with her partner	124/605 (41 %)	95/274 (35 %)	465/504 (92 %)	305/427 (71 %)	1.30 (1.03–1.65)
Okay for a woman to ask her husband to use a condom	-	-	401/504 (80 %)	242/427 (57 %)	1.41 (1.18–1.69)
*Broader gender attitudes:*					
Others in community would respect a man who made decisions jointly with his wife	-	-	385/504 (76 %)	227/427 (53 %)	1.49 (0.91–2.44)
Man’s role to decide if his wife can work	-	-	226/504 (45 %)	288/427 (67 %)	0.67 (0.54–0.81)
*Behaviours:*					
Drunk at least once a month	-	-	67/502 (13 %)	56/422 (13 %)	0.98 (0.56–1.70)
Woman experiencing (man perpetrating) violence who has told someone	78/132 (59 %)	54/112 (48 %)	184/271 (68 %)	170/301 (56 %)	1.22 (0.81–1.85)

^a^Question wording/item construction changed between baseline and follow-up to improve face validity - those baseline measures closest to the follow-up outcomes are presented here to assess underlying intervention/control community comparability, but baseline/follow-up comparisons are not possible

^b^Risk ratios calculated at the cluster-level, adjusted for community-pair, and weighted according to the number of observations per village. Adjusted risk ratios generated on the basis of expected number of events from a logistic regression model on individual data with independent variables including age and marital status

^c^ Mean number of respondents per EA = 28.0 (range 18–35)

^*^χ^2^ p-value <0.005

**Table 4:** Estimates of intervention effect on potential mediators among men, comparing outcome in intervention versus control communities

	Baseline		Follow-up		
	Intervention	Control	Intervention	Control	aRR^a^ (95 % CI)
COMMUNITY LEVEL^b^	*EA-level mean risk % (sd) n = 16*	*EA-level mean risk % (sd) n = 16*	*Mean difference (95 % CI)*	*EA-level mean risk % (sd) n = 24*	*Mean difference (95 % CI)*
*Community responses to prevent violence:*					
Okay for others in community to intervene if they know IPV is occurring	-	-	92.5 (6.6)	42.8 (11.9)	47.6 (21.9–73.3)
People who have witnessed/heard violence who have responded appropriately	-	-	62.8 (15.1)	26.1 (10.7)	33.3 (−4.0–70.6)
*Norms around violence:*					
Acceptable for a man to use violence against his partner	27.7 (17.2)	25.2 (15.8)	7.3 (5.8)	85.6 (6.6)	−75.5 (−92.4– −58.7)
*Norms around women’s control over sex:*					
Acceptable for a woman to refuse sex with her partner	53.5 (18.3)	55.6 (14.0)	97.5 (2.6)	75.4 (12.9)	23.0 (1.0–45.0)
Okay for a woman to ask her husband to use a condom	-	-	88.6 (12.2)	43.6 (8.8)	41.8 (17.8–65.8)
*Broader gender norms:*					
Others in community would respect a man who made decisions jointly with his wife	-	-	88.9 (10.5)	38.9 (10.9)	48.3 (29.7–66.9)
Man’s role to decide if his wife can work	-	-	14.8 (10.6)	83.6 (5.4)	−67.0 (−82.1– −51.9)
RELATIONSHIP LEVEL (PARTNERED IN PAST YEAR)	*n/N (%)*	*n/N (%)*	*n/N (%)*	*n/N (%)*	*aRR (95 % CI)*
*Communication:*					
Discuss things that happen in day	275/313 (88 %)	292/335 (87 %)	523/545 (96 %)	318/434 (73 %)	1.30 (0.98–1.72)
Discuss worries	270/313 (86 %)	294/335 (88 %)	525/545 (96 %)	326/434 (75 %)	1.28 (1.00–1.64)
Discuss what you both like during sex	-	-	481/544 (88 %)	226/434 (52 %)	1.70 (1.22–2.37)
Appreciate work partner does around house	-	-	385/409 (94 %)	231/326 (71 %)	1.32 (1.04–1.69)
Appreciate work partner does outside house	-	-	228/283 (81 %)	128/244 (52 %)	1.61 (1.04–2.50)
*Power dynamics:*					
Joint decision making	208/234 (89 %)	229/262 (87 %)	378/443 (85 %)	165/356 (46 %)	1.90 (1.28–2.80)
Man helps around house	180/304 (59 %)	214/330 (65 %)	396/411 (96 %)	229/326 (70 %)	1.42 (0.98–2.05)
Woman refused a job because husband doesn’t want her to work	26/313 (8 %)	26/335 (8 %)	27/506 (5 %)	123/410 (30 %)	0.12 (0.02–0.89)
Woman participated in deciding how household finances spent	234/306 (76 %)^*^	271/327 (83 %)^*^	416/449 (93 %)	218/345 (63 %)	1.48 (1.11–1.97)
*Additional sex partners:*					
Concurrent partners	109/270 (40 %)	105/284 (37 %)	139/508 (27 %)	177/397 (45 %)	0.60 (0.37–0.97)
Male partner often suspicious that female partner is unfaithful	-	-	76/620 (12 %)	221/525 (42 %)	0.19 (0.02–1.60)
*Relationship dissolution:*					
Separated/divorced in past year	3/307 (1 %)	5/330 (2 %)	7/545 (1 %)	12/435 (3 %)	0.52 (0.15–1.83)
INDIVIDUALS (PARTNERED IN PAST YEAR)					
*Attitudes around violence:*					
Acceptable for a man to use violence against his partner	81/313 (26 %)	83/335 (25 %)	119/624 (19 %)	454/525 (86 %)	0.14 (0.02–1.11)
Okay for a woman to tell others if she is experiencing violence	-	-	571/624 (92 %)	221/525 (42 %)	2.24 (1.39–3.61)
*Attitudes towards women’s control over sex:*					
Acceptable for a woman to refuse sex with her partner	164/313 (52 %)	184/335 (55 %)	608/624 (97 %)	400/525 (76 %)	1.30 (0.96–1.78)
Okay for a woman to ask her husband to use a condom	-	-	536/624 (86 %)	245/525 (47 %)	1.86 (1.28–2.70)
*Broader gender attitudes:*					
Others in community would respect a man who made decisions jointly with his wife	-	-	541/624 (87 %)	202/525 (38 %)	2.27 (1.53–3.36)
Man’s role to decide if his wife can work	-	-	192/624 (31 %)	448/525 (85 %)	0.27 (0.06–1.25)
*Behaviours:*					
Drunk at least once a month	92/311 (30 %)	110/329 (33 %)	162/619 (26 %)	200/525 (38 %)	0.69 (0.38–1.27)
Woman experiencing (man perpetrating) violence who has told someone	39/105 (37 %)	50/116 (43 %)	101/181 (56 %)	172/452 (38 %)	1.50 (0.80–2.83)

^a^Risk ratios calculated at the cluster-level, adjusted for community-pair, and weighted according to the number of observations per village. Adjusted risk ratios generated on the basis of expected number of events from a logistic regression model on individual data with independent variables including age and marital status

^b^ Mean number of respondents per EA = 28.0 (range 18–35)

^*^χ^2^ p-value <0.005

As reported in the main trial paper, the relative risk of past year experience of physical IPV among women was 52 % lower in intervention communities compared to control communities at follow-up (aRR 0.48, 95 % CI 0.16–1.39) [27]. Relative risk of men’s past year perpetration of IPV (anonymously reported) was 61 % lower in intervention communities compared to control communities (aRR 0.39, 95 % CI 0.20–0.73).

### Which mediating factors played a role in reducing IPV?

Intervention impacts on potential mediators are presented in Tables 3 (women) and 4 (men), and the relationships between these mediators and IPV in Table 5. Table 6 shows the impact of SASA! on IPV experience (women)/perpetration (men), after adjustment for potential mediators.

**Table 5:** Associations between potential mediators and past year experience (women)/perpetration (men) of physical IPV among respondents partnered in past year

	Women - aRR^a^ (95 % CI) for past year experience of IPV	Men - aRR^a^ (95 % CI) for past year perpetration of IPV
COMMUNITY LEVEL	*aRR of IPV for every 10 % change in community-prevalence of mediator*	*aRR of IPV for every 10 % change in community-prevalence of mediator*
*Community responses to prevent violence:*		
Okay for others in community to intervene if they know IPV is occurring	0.94 (0.79–1.13)	0.88 (0.74–1.04)
People who have witnessed/heard violence who have responded appropriately	0.98 (0.87–1.10)	0.91 (0.80–1.04)
*Norms around violence:*		
Acceptable for a man to use violence against his partner	1.35 (1.18–1.54)	1.18 (0.99–1.40)
*Norms around women’s control over sex:*		
Acceptable for a woman to refuse sex with her partner	0.85 (0.68–1.06)	1.13 (0.90–1.43)
Okay for a woman to ask her husband to use a condom	0.76 (0.61–0.95)	0.90 (0.74–1.09)
*Broader gender norms:*		
Others in community would respect a man who made decisions jointly with his wife	0.79 (0.67–0.94)	0.91 (0.79–1.06)
Man’s role to decide if his wife can work	1.34 (1.17–1.54)	1.16 (1.00–1.35)
RELATIONSHIP LEVEL (PARTNERED IN PAST YEAR)	*aRR of IPV in individuals with versus without mediator*	*aRR of IPV in individuals with versus without mediator*
*Communication:*		
Discuss things that happen in day	0.36 (0.23–0.56)	0.99 (0.58–1.70)
Discuss worries	0.31 (0.18–0.54)	0.95 (0.63–1.43)
Discuss what you both like during sex	0.42 (0.28–0.61)	0.67 (0.41–1.08)
Appreciate work partner does around house	0.44 (0.28–0.69)	0.56 (0.35–0.90)
Appreciate work partner does outside house	0.46 (0.27–0.80)	0.53 (0.34–0.80)
*Power dynamics:*		
Joint decision making	0.27 (0.16–0.47)	0.61 (0.39–0.96)
Man helps around house	0.63 (0.39–1.03)	0.59 (0.35–0.98)
Woman refused a job because husband doesn’t want her to work	4.65 (2.59–8.36)	2.78 (1.70–4.56)
Woman participated in deciding how household finances spent	0.38 (0.25–0.58)	0.84 (0.58–1.23)
*Additional sex partners:*		
Concurrent partners	1.93 (0.78–4.77)	2.79 (2.00–3.90)
Male partner often suspicious that female partner is unfaithful	6.35 (3.73–10.80)	4.51 (3.31–6.13)
*Relationship dissolution:*		
Separated/divorced in past year	4.25 (1.49–12.13)	2.13 (0.42–10.88)
INDIVIDUALS (PARTNERED IN PAST YEAR)		
*Attitudes around violence:*		
Acceptable for a man to use violence against his partner	2.45 (1.65–3.66)	2.17 (1.43–3.28)
Okay for a woman to tell others if she is experiencing violence	0.73 (0.49–1.11)	0.52 (0.32–0.85)
*Attitudes towards women’s control over sex:*		
Acceptable for a woman to refuse sex with her partner	0.46 (0.30–0.70)	0.92 (0.55–1.56)
Okay for a woman to ask her husband to use a condom	0.51 (0.34–0.76)	0.85 (0.60–1.19)
*Broader gender attitudes:*		
Others in community would respect a man who made decisions jointly with his wife	0..39 (0.24–0.63)	0.60 (0.43–0.83)
Man’s role to decide if his wife can work	2.25 (1.38–3.69)	1.92 (1.29–2.85)
*Behaviours:*		
Drunk at least once a month	1.61 (0.89–2.93)	2.33 (1.82–3.00)

^a^Risk ratios adjusted for site-pair, intervention arm, age, marital status, education and childhood experiences of abuse

**Table 6:** SASA! impact on women’s past year experience/men’s past year perpetration of physical IPV, after adjustment for potential mediators

Mediator adjusted for:	aRR^a^ (95 % CI) for SASA! impact on women’s experience of IPV	% change in aRR after addition of mediator	aRR^a^ (95 % CI) for SASA! impact on men’s perpetration of IPV	% change in aRR after addition of mediator
	*n* = 875		*n* = 1108	
Model without mediators	0.44 (0.30–0.64)	N/A	0.45 (0.30–0.70)	N/A
COMMUNITY LEVEL				
*Community responses to prevent violence:*				
Okay for others in community to intervene if they know IPV is occurring	0.44 (0.29–0.68)	0 %	0.66 (0.34–1.27)	38 %
People who have witnessed/heard violence who have responded appropriately	0.43 (0.30–0.63)	−2 %	0.52 (0.31–86)	13 %
*Norms around violence:*				
Acceptable for a man to use violence against his partner	0.83 (0.50–1.38)	70 %	0.97 (0.40–2.39)	95 %
*Norms around women’s control over sex:*				
Acceptable for a woman to refuse sex with her partner	0.57 (0.34–0.96)	23 %	0.41 (0.26–0.64)	−7 %
Okay for a woman to ask her husband to use a condom	0.62 (0.39–0.99)	32 %	0.56 (0.30–1.02)	20 %
*Broader gender norms:*				
Others in community would respect a man who made decisions jointly with his wife	0.64 (0.40–1.01)	36 %	0.58(0.34–1.00)	24 %
Man’s role to decide if his wife can work	0.70 (0.48–1.03)	46 %	0.82 (0.42–1.59)	67 %
RELATIONSHIP LEVEL (PARTNERED IN PAST YEAR)				
*Communication:*				
Discuss things that happen in day	0.51 (0.35–0.74)	13 %	0.46 (0.30–0.69)	2 %
Discuss worries	0.51 (0.36–0.73)	13 %	0.47 (0.31–0.70)	4 %
Discuss what both like during sex	0.53 (0.36–0.77)	16 %	0.49 (0.32–0.75)	7 %
Appreciate work partner does around house	0.46 (0.32–0.65)	4 %	0.48 (0.32–0.72)	5 %
Appreciate work partner does outside house	0.45 (0.31–0.65)	2 %	0.48 (0.32–0.71)	5 %
*Power dynamics:*				
Joint decision making	0.52 (0.37–0.74)	14 %	0.49 (0.34–0.72)	7 %
Man helps around house	0.47 (0.32–0.67)	5 %	0.49 (0.32–0.73)	7 %
Woman refused a job because husband doesn’t want her to work	0.48 (0.34–0.70)	7 %	0.51 (0.34–0.76)	11 %
Woman participated in deciding how household finances spent	0.47 (0.34–0.66)	5 %	0.47 (0.32–0.70)	4 %
*Additional sex partners:*				
Concurrent partners	-	-	0.49 (0.34–0.73)	7 %
Male partner often suspicious that female partner is unfaithful	0.54 (0.38–0.76)	18 %	0.57 (0.40–0.81)	22 %
INDIVIDUALS (PARTNERED IN PAST YEAR)				
*Attitudes around violence:*				
Acceptable for a man to use violence against his partner	0.53 (0.36–0.80)	16 %	0.68 (0.43–1.09)	42 %
Okay for a woman to tell others if she is experiencing violence	0.46 (0.32–0.67)	4 %	0.57 (0.37–0.90)	22 %
*Attitudes towards women’s control over sex:*				
Acceptable for a woman to refuse sex with her partner	0.54 (0.37–0.77)	18 %	0.48 (0.31–0.73)	5 %
Okay for a woman to ask her husband to use a condom	0.51 (0.34–0.76)	13 %	0.47 (0.31–0.72)	4 %
*Broader gender attitudes:*				
Others in community would respect a man who made decisions jointly with his wife	0.53 (0.36–0.78)	16 %	0.54 (0.36–0.83)	16 %
Man’s role to decide if his wife can work	0.51 (0.35–0.72)	13 %	0.58 (0.37–0.90)	24 %
*Behaviours:*				
Drunk at least once a month	-	-	0.47 (0.33–0.69)	4 %

^a^Adjusted risk ratios calculated using modified poisson regression with cluster robust standard errors, and adjusted for site-pair, age, marital status and EA-level baseline prevalence of IPV

#### Community-level mediators

Higher proportions of both men and women in SASA! communities compared to control communities reported responding appropriately if they had seen or heard IPV occurring in the past year. However, improved community response only appeared to be associated (non-significantly) with reduced risk of perpetration of IPV among men, not experience of IPV among women. In line with this, only among men was there was a slight attenuation of intervention effect on IPV when community response was included in the model (13 % reduction in effect size). The same picture was true for community attitudes towards the acceptability of intervening in cases of abuse - the mediating effect (this time stronger) only seen in relation to men’s perpetration of IPV (38 % reduction in effect size), not women’s experience.

SASA! was associated with a reduction in community-level acceptance of men using violence against their partners, a factor which in turn is associated with a lower risk of both IPV experience among women and IPV perpetration among men. Reduced acceptance of IPV at the community-level appears to have been a very important mediator of intervention impact on IPV among both women and men - inclusion of community attitudes towards violence in the model led to a 70 % reduction in effect size for women’s experience of IPV, and almost total attenuation of effect for men’s perpetration.

SASA! communities were also more accepting than control communities of a woman’s right both to refuse sex and to ask her husband to use a condom. The latter was more strongly related to reduced IPV risk, especially among women, and was also the more important mediator of intervention effect on IPV. There was a 32 % reduction in effect size for women, and a 20 % reduction in effect size for men, after acceptability of requesting condom use was included in the model.

SASA! led to increased community acceptance of joint decision making between partners, and decreased acceptance that it is a man’s role to decide whether or not his wife can work. Both appear to be potential mediators of intervention impact on IPV, with the strongest attenuation of intervention impact seen in relation to community rejection of the notion that it is a man’s role to decide if his wife can work (46 % reduction in effect size for women; 67 % reduction in effect size for men).

#### Relationship-level mediators

SASA! was associated with better communication between partners, as reported by both men and women (discussing things that happen to them both in the day, discussing worries, discussing what they both like during sex, and showing appreciation for work their partner does around the house). All of these indicators were in turn significantly associated with reduced IPV experience among women, though only appreciation of a partner’s work was significantly associated with IPV perpetration among men. Improved communication appears to play a more important mediating role in intervention effect on IPV experience among women than it does in relation to IPV perpetration among men. The largest attenuation of effect among women was seen when discussion of what both partners like during sex was included in the model (16 % reduction).

Men and women in SASA! communities were also more likely to be in relationships characterised by more progressive power dynamics (more joint decision-making, the man helping around the house, the woman not made to refuse a job because her husband didn’t want her to work, and the woman participating in deciding how household finances are spent). Indicators of greater equality within the relationship were significantly associated with reduced risk of IPV experience among women, and reduced perpetration among men. However, they emerged as only moderate mediators of intervention effect on IPV - the strongest attenuation of effect in women seen when joint decision-making was included in the model (14 % reduction in effect size), and the strongest in men when the indicator of his partner refusing a job was included in the model (11 % reduction in effect size).

Men in SASA! communities were less likely than their control counterparts to report having had concurrent sexual partners in the past year. While sexual concurrency by the man is statistically significantly associated with men’s reported perpetration of IPV, it does not appear to be a mediator of intervention effect on perpetration of IPV.

Women overall reported low levels of sexual concurrency, with no difference observed between intervention and control communities. However, both women and men in SASA! communities were less likely than their control counterparts to report that the man was often suspicious that his female partner was unfaithful. Reduced suspicion emerges as an important mediator of intervention effect on both women’s IPV experience and men’s IPV perpetration (18 % reduction in effect size for women; 22 % reduction for men).

Relationship dissolution was slightly lower in SASA! communities than it was in control communities (though not significantly so), suggesting that SASA!’s effect on violence was not due to increased numbers of women leaving abusive relationships.

#### Individual-level mediators - women

Women in SASA! communities were less likely than their control counterparts to be accepting of a man’s use of violence against his female partner (an attitude associated with increased risk of IPV), and more likely to think that it was okay for a woman to tell others if she was experiencing violence at the hands of her partner (an attitude associated with decreased risk of IPV). Of these two individual-level indicators, only attitudes accepting of violence emerged as a potentially important mediator of intervention effect on IPV, with its inclusion in the model resulting in a 16 % reduction in effect size.

Women in SASA! communities were also more likely to believe that it was acceptable for a woman to refuse sex with her partner if she did not feel like it, and that it was okay for a woman to ask her husband to use a condom. Both of these were associated with a significant reduction in risk of IPV experience, and also emerged as potential mediators of intervention effect on IPV (attenuating effect size by 18 and 13 % respectively when included in the model).

A higher proportion of women in SASA! communities reported believing that others in the community would respect a man who made decisions jointly with his wife (a perception associated with decreased IPV risk). Fewer were of the view that it is a man’s role to decide if his wife can work (a view associated with increased IPV risk). Both indicators emerged as potential mediators of intervention effect on IPV, attenuating effect by 16 and 13 % respectively.

SASA! did not impact on women’s drinking behaviour.

#### Individual-level mediators – men

Men in SASA! communities were less likely than their control counterparts to be accepting of violence (an attitude associated with increased risk of perpetrating IPV), and more likely to believe that it was okay for a woman to tell others if she was experiencing violence (an attitude associated with decreased risk of perpetrating IPV). Both indicators were important mediators of intervention effect on perpetration of IPV, the former leading to a 42 % reduction in effect size when included in the model, and the latter a 24 % reduction.

Men in SASA! communities were also more likely to believe that it was acceptable for a woman to refuse sex with her partner, and that it was okay for a woman to ask her husband to use a condom. However, neither was associated with risk of IPV perpetration, and consequently neither was shown to mediate intervention effect on perpetration of IPV.

A higher proportion of men in SASA! communities believed that others in the community would respect a man who made decisions jointly with his wife (a perception associated with a decreased risk of perpetrating IPV). Fewer held the view that it is a man’s role to decide if his wife can work (a view associated with an increased risk of perpetrating IPV). Both indicators emerged as potentially important mediators of intervention effect on IPV perpetration - their inclusion in the model attenuating intervention effect by 16 and 24 % respectively.

While a slight (though statistically non-significant) decrease in frequent drunkenness was observed among men in SASA! communities, and despite the fact that frequent drunkenness is strongly associated with increased risk of IPV perpetration, changes in drinking behaviour do not appear to have been mediators of intervention effect on perpetration of IPV.

## Discussion

This analysis has identified a number of potentially important pathways through which SASA! worked to prevent physical IPV against women. Pathways include factors operating at the community-, relationship- and individual-level, and reinforce findings from qualitative research conducted with SASA! community members [34].

At the community-level, changes in normative attitudes, particularly those around the acceptability of violence and more progressive gender relations, appear to have played a hugely significant mediating role in preventing IPV. Reductions in the acceptability of violence explain most of the intervention effect seen in women and almost all of the effect seen in men. The extent of the mediating role of normative attitudes, especially among men, suggests peer pressure and a perceived threat of sanctions as potentially important mechanisms for preventing perpetration of IPV.

Community responses to IPV were observed to somewhat mediate the impact of SASA! on male perpetration of IPV, but not on female experience of IPV. It is plausible that community responses on the part of other men are more effective at producing fear of sanctions among men (and thereby discouraging perpetration) than other women’s responses are at helping women out of abusive situations. There is also a possible two-way relationship between community response and individual risk of IPV. High rates of IPV within a community could motivate and prime community members to mount a better response to the violence, thereby causing us to underestimate the role of community response as a mediator in IPV reductions.

At the relationship-level, several indicators of improved communication and more equal power dynamics appear to play a moderate mediating role in intervention effect on IPV experience among women. Their mediating role was smaller in relation to men’s perpetration. Potential mechanisms of mediation include reductions in stressful situations and miscommunications that might trigger relationship conflict and violence [34].

Interestingly, suspicion by the man that his female partner is unfaithful was the most important relationship-level mediator of intervention effect on both women’s experience and men’s perpetration of IPV. This suggests that efforts to reduce levels of suspicion within relationships - for example through encouraging improved communication, or challenging gender norms that make it unacceptable for a woman to refuse sex with her husband or seek work outside the home without arousing suspicion - could form a potentially important part of violence prevention strategies.

At the individual-level, it is interesting to note that male attitudes towards the acceptability of IPV are much more important mediators of intervention effect on IPV perpetration, than female attitudes are in relation to IPV experience. This is in line with evidence from other studies [19], and is not surprising. While a man’s attitudes can directly influence whether or not he uses violence, a woman’s attitudes can impact on her own experiences of IPV only indirectly, through influencing her choice of partner or her motivation to leave an abusive partner - the latter reaction does not appear to have become more common in SASA! communities.

Among men, attitudes towards the acceptability of IPV appear to be more influential mediators of intervention effect than attitudes towards broader gender norms. This supports the idea that reductions in broad measures of gender inequality may not be sufficient in themselves to prevent violence, if specific attitudes towards the acceptability of violence against women are not also directly addressed and challenged. The finding that men’s individual-level attitudes towards a woman’s right to refuse sex do not appear to play a role in IPV reduction, also suggests that the issue of sexual coercion within a partnership might not always be strongly linked to use of physical violence.

This study has many strengths, not least that the data come from a cluster randomised trial which allows us to compare intervention and control communities and thus identify mediating factors that are associated with *reductions* in violence, not just prevalence of violence. Nevertheless, there are limitations to this analysis. As with other violence research studies, our analysis is of self-reported data which may be prone to respondent or recall bias. Another limitation is that the data are cross-sectional, meaning it is not possible to determine whether observed associations are causal and, if they are, what the direction of that causal association is. The randomised design and ITT analysis, along with baseline data showing intervention and control communities to be highly comparable prior to intervention implementation, lend support to the interpretation that SASA! positively impacted on the mediating variables. However, we cannot rule out a loop of causality in the latter end of the causal pathway, whereby the change in a mediator is brought about by a reduction in IPV rather than the reverse scenario. Prior to conducting any analysis, we laid out theoretically and empirically grounded plausible pathways of effect in a conceptual framework. Our analysis and interpretation thus reflects our *a priori* suppositions about causality and directions of effect, rather than definitively testing a causal pathway. Another likely source of endogeneity is that the role of each mediator may have been positively confounded by other variables not included in the model (including other potential mediators with which it is correlated). As already stated, due to the complex web of causation invoked by an intervention such as SASA!, involving a multitude of closely interrelated social phenomena, our analysis attempts to identify which types of mediators *play a role in* reducing IPV rather than producing precise estimates of the proportion of intervention effect that can be attributed to each. While it is possible that the role of certain mediators has been exaggerated, confounding is unlikely to wholly explain the large attenuations of effect observed when, for example, community level attitudes are included in the model of intervention effect on IPV.

A further constraint that we faced in the analysis is that, while we looked at pathways to both IPV experience (women) and IPV perpetration (men), our data did not pertain to men and women in the same relationships, nor living within the same immediate localities. We could not therefore directly explore the mediating influences of men’s factors (including community norms among men) on women’s experiences of IPV, or women’s factors (including community norms among women) on men’s perpetration of IPV. Nevertheless, the broad range of mediators included in the analysis still allows us to gain insights into diverse pathways through which the intervention may have brought about reductions in IPV.

Despite limitations, this study provides major new insights to the field of violence prevention research. One interesting finding is that attitudes are similarly related to IPV risk in both intervention and control communities. This suggests that SASA! has not only managed to change what people say they believe, but has changed attitudes in a meaningful way that relates to behaviours. Evidence that such change can occur over a relatively short period of programming offers validation to the approaches of interventions such as SASA! which aim to prevent violence through norm change.

Most importantly, the study sheds light on key pathways of effect that violence prevention interventions can and should exploit. At the relationship-level, an important insight is the role that reduced suspicion of infidelity within the relationship may play in mediating intervention effect on IPV. The finding that men’s attitudes play more of a mediating role than women’s attitudes points to the importance of working with both men and women, in contrast to approaches which work solely with women. Perhaps most noteworthy is the finding that community-level factors, in particular norms relating to the acceptability of a man’s use of violence against his partner, are the major mediators of intervention effect on both female experience and male perpetration of IPV. They appear to play a more significant mediating role than changes at either the relationship- or individual-level. To our knowledge, such a finding has not been previously demonstrated in relation to a violence prevention intervention study. It highlights the imperative of addressing the underlying contexts in which IPV occurs, and lends strong support for the more widespread adoption of community-level approaches to preventing violence.

## Conclusion

The SASA! study was the first CRT in sub-Saharan Africa to assess the community-level impact of a violence prevention programme. Results suggest not only that community-level violence prevention is possible over a relatively short time-frame, but that community-level norm change may in fact be the most effective means of achieving reductions in IPV risk. They also demonstrate the importance of working with both men and women to achieve reductions in IPV risk, with changes to individual-level attitudes and relationship dynamics as reported by both men and women emerging as pathways through which violence was reduced. Overall, these findings have important implications for violence prevention programming - while programmes have often focused on changing attitudes among individual programme recipients, greater reductions in risk might be achieved by changing pervasive norms in the wider community. As testimony to its success, SASA! is currently being replicated in 14 countries - the core systematic approach to tackling the underlying community-level drivers of violence preserved amidst local adaptations to materials, activities, activity settings and key personnel. More research and investment is now urgently needed for the further development of community-level interventions to prevent violence, and to better understand and support their effective replication and scale-up.

## Availability of supporting data

Data from the follow-up survey and limited data from the baseline survey can be requested from the London School of Hygiene and Tropical Medicine Data Compass: http://datacompass.lshtm.ac.uk/19/

## Additional files

Additional file 1: Intervention logic model. Detailed description of intervention logic model. (PPT 238 kb) Additional file 2: Trial profile. Diagram showing the flow of communities and participants through the trial. (PPTX 72 kb) Additional file 3: Descriptive data on study sites and respondents. Descriptive data comparing intervention and control communities at baseline and follow-up. (DOCX 16 kb)

## Abbreviations

aRR: adjusted risk ratio
CA: community activist (in the SASA! Intervention)
CEDOVIP: Centre for Domestic Violence Prevention
CI: confidence interval
CRT: cluster randomized trial
EA: enumeration area
IPV: intimate partner violence
