# Supplementary material for: Ecological pathways to prevention: How does the SASA! community mobilisation model work to prevent physical intimate partner violence against women?
Source: BMC Public Health. 2016 Apr 16;16:339. doi: 10.1186/s12889-016-3018-9 (PMC4833941; doi:10.1186/s12889-016-3018-9)
Supplement: Additional file 1: — Intervention logic model. Detailed description of intervention logic model. (PPT 238 kb) [file 12889_2016_3018_MOESM1_ESM.ppt]

## Slide 1
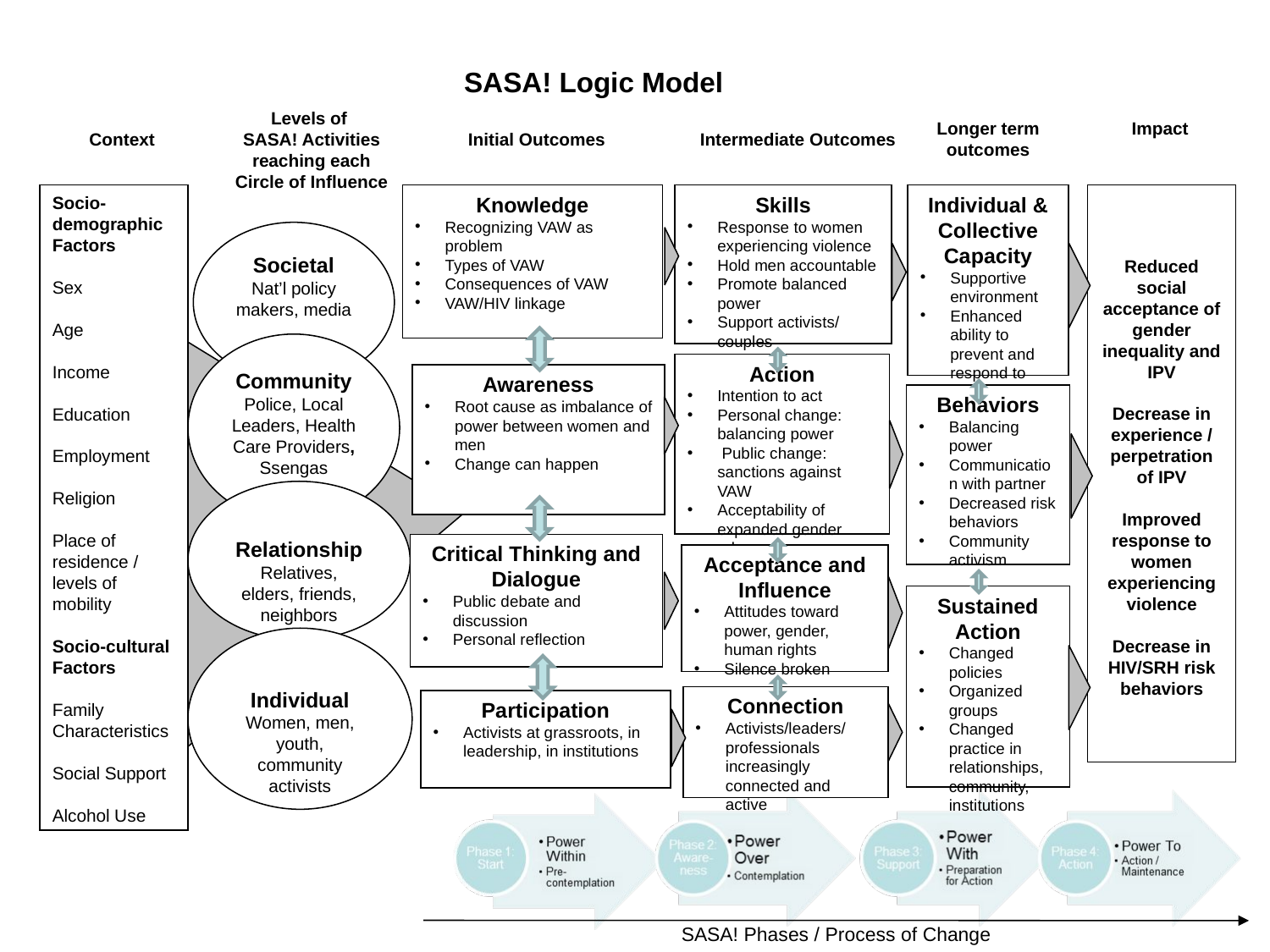

SASA! Logic Model
Levels of
SASA! Activities reaching each Circle of Influence
Longer term outcomes
Impact
Context
Initial Outcomes
Intermediate Outcomes
Socio-demographic Factors
Sex
Age
Income
Education
Employment
Religion
Place of residence / levels of mobility
Socio-cultural Factors
Family Characteristics
Social Support
Alcohol Use
Knowledge
Recognizing VAW as problem
Types of VAW
Consequences of VAW
VAW/HIV linkage
Skills
Response to women experiencing violence
Hold men accountable
Promote balanced power
Support activists/ couples
Reduced social acceptance of gender inequality and IPV
Decrease in experience / perpetration of IPV
Improved response to women experiencing violence
Decrease in HIV/SRH risk behaviors
Societal
Nat’l policy makers, media
Community
Police, Local Leaders, Health Care Providers, Ssengas
Action
Intention to act
Personal change: balancing power
 Public change: sanctions against VAW
Acceptability of expanded gender roles
Awareness
Root cause as imbalance of power between women and men
Change can happen
Behaviors
Balancing power
Communication with partner
Decreased risk behaviors
Community activism
Relationship
Relatives, elders, friends, neighbors
Critical Thinking and Dialogue
Public debate and discussion
Personal reflection
Acceptance and Influence
Attitudes toward power, gender, human rights
Silence broken
Sustained Action
Changed policies
Organized groups
Changed practice in relationships, community, institutions
Individual
Women, men, youth, community activists
Connection
Activists/leaders/professionals increasingly connected and active
Participation
Activists at grassroots, in leadership, in institutions
Individual & Collective Capacity
Supportive environment
Enhanced ability to prevent and respond to VAW
SASA! Phases / Process of Change
