# Supplementary material for: Ecological pathways to prevention: How does the SASA! community mobilisation model work to prevent physical intimate partner violence against women?
Source: BMC Public Health. 2016 Apr 16;16:339. doi: 10.1186/s12889-016-3018-9 (PMC4833941; doi:10.1186/s12889-016-3018-9)
Supplement: Additional file 2: — Trial profile. Diagram showing the flow of communities and participants through the trial. (PPTX 72 kb) [file 12889_2016_3018_MOESM2_ESM.pptx]

## Slide 1
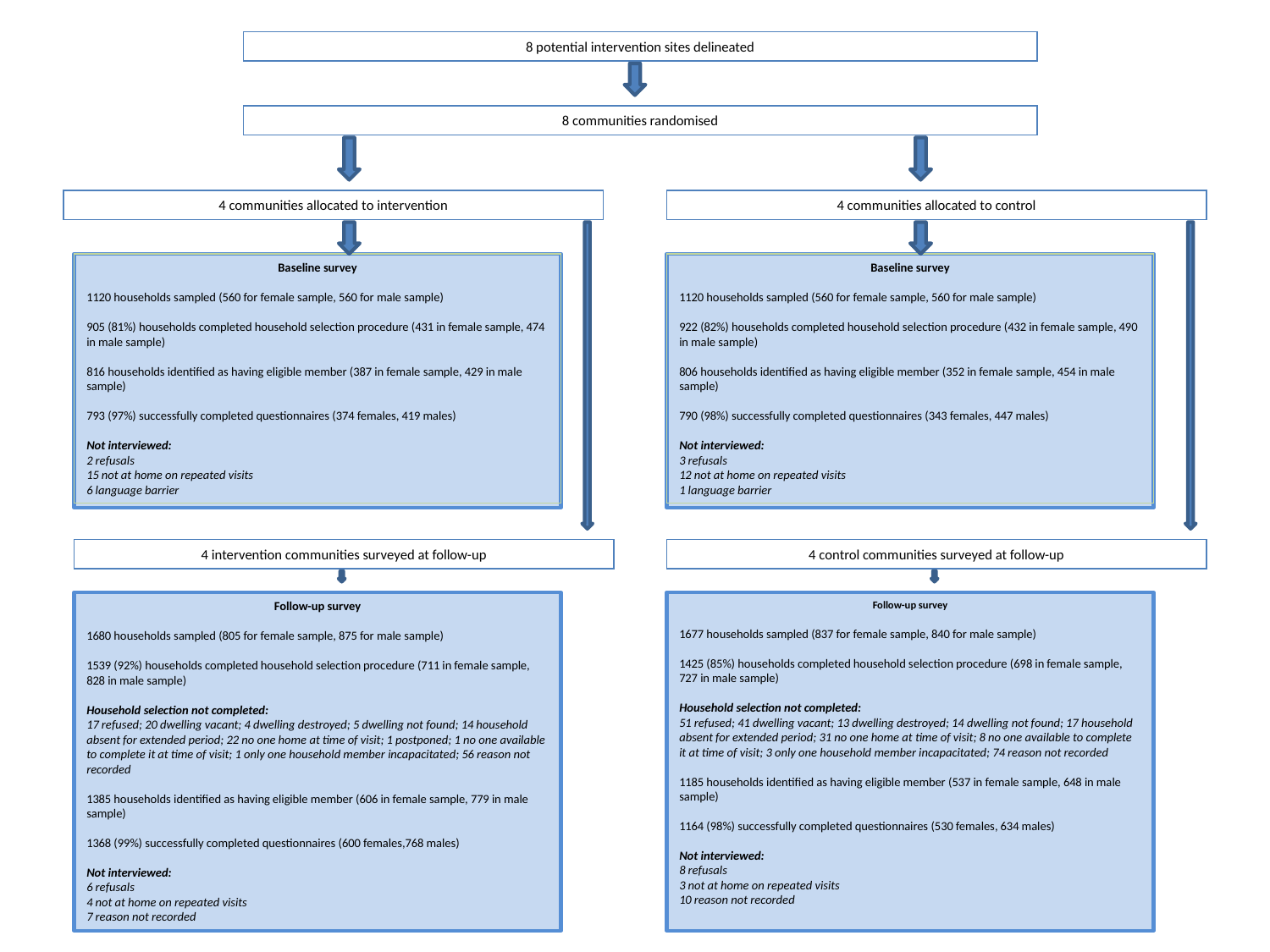

8 potential intervention sites delineated
8 communities randomised
4 communities allocated to intervention
4 communities allocated to control
Baseline survey
1120 households sampled (560 for female sample, 560 for male sample)
905 (81%) households completed household selection procedure (431 in female sample, 474 in male sample)
816 households identified as having eligible member (387 in female sample, 429 in male sample)
793 (97%) successfully completed questionnaires (374 females, 419 males)
Not interviewed:
2 refusals
15 not at home on repeated visits
6 language barrier
Baseline survey
1120 households sampled (560 for female sample, 560 for male sample)
922 (82%) households completed household selection procedure (432 in female sample, 490 in male sample)
806 households identified as having eligible member (352 in female sample, 454 in male sample)
790 (98%) successfully completed questionnaires (343 females, 447 males)
Not interviewed:
3 refusals
12 not at home on repeated visits
1 language barrier
4 intervention communities surveyed at follow-up
4 control communities surveyed at follow-up
Follow-up survey
1680 households sampled (805 for female sample, 875 for male sample)
1539 (92%) households completed household selection procedure (711 in female sample, 828 in male sample)
Household selection not completed:
17 refused; 20 dwelling vacant; 4 dwelling destroyed; 5 dwelling not found; 14 household absent for extended period; 22 no one home at time of visit; 1 postponed; 1 no one available to complete it at time of visit; 1 only one household member incapacitated; 56 reason not recorded
1385 households identified as having eligible member (606 in female sample, 779 in male sample)
1368 (99%) successfully completed questionnaires (600 females,768 males)
Not interviewed:
6 refusals
4 not at home on repeated visits
7 reason not recorded
Follow-up survey
1677 households sampled (837 for female sample, 840 for male sample)
1425 (85%) households completed household selection procedure (698 in female sample, 727 in male sample)
Household selection not completed:
51 refused; 41 dwelling vacant; 13 dwelling destroyed; 14 dwelling not found; 17 household absent for extended period; 31 no one home at time of visit; 8 no one available to complete it at time of visit; 3 only one household member incapacitated; 74 reason not recorded
1185 households identified as having eligible member (537 in female sample, 648 in male sample)
1164 (98%) successfully completed questionnaires (530 females, 634 males)
Not interviewed:
8 refusals
3 not at home on repeated visits
10 reason not recorded
