# Supplementary material for: Ecological pathways to prevention: How does the SASA! community mobilisation model work to prevent physical intimate partner violence against women?
Source: BMC Public Health. 2016 Apr 16;16:339. doi: 10.1186/s12889-016-3018-9 (PMC4833941; doi:10.1186/s12889-016-3018-9)
Supplement: Additional file 3: — Descriptive data on study sites and respondents. Descriptive data comparing intervention and control communities at baseline and follow-up. (DOCX 16 kb) [file 12889_2016_3018_MOESM3_ESM.docx]

**Appendix 3: Descriptive data on study sites and respondents**

**Appendix 3a:** Site-level characteristics at baseline and follow-up

| **Site-level characteristics** | Baseline | | Follow-up | |
| --- | --- | --- | --- | --- |
|  | intervention | Control | intervention | Control |
|  | mean^a^ (range) | mean^a^ (range) | mean^a^ (range) | mean^a^ (range) |
|  |  |  |  |  |
| Number of sites | 4 | 4 | 4 | 4 |
| Number of CAs per site | two sites with 8 (4 female, 4 male) | two sites with 8 (4 female, 4 male) | two sites with 8 (4 female, 4 male); | two sites with 8 (4 female, 4 male); |
|  | two sites with 16 (8 female, 8 male) | two sites with 16 (8 female, 8 male) | two sites with 16 (8 female, 8 male) | two sites with 16 (8 female, 8 male) |
| Number of enumeration areas sampled per site^b^ | 8 | 8 | 8 in two sites, 16 in two sites | 8 in two sites, 16 in two sites |
| Number of households per site (in sampling frame) | 1,866 (852 to 2,648) | 1,367 (974 to 1,829) | 3,190 (1,866 to 4,465) | 1,811 (1,444 to 2,526) |
| % of households with electricity | 74 (65 to 80) | 79 (67 to 89) | 85 (82 to 89) | 85 (83 to 86) |
| % of households where main drinking water source is a public tap | 63 (52 to 80) | 68 (57 to 80) | 65 (42 to 80) | 64 (53 to 80) |
| % of households using traditional pit toilet/latrine | 63 (57 to 74) | 60 (55 to 64) | 57 (49 to 67) | 54 (45 to 61) |
| % of households living in rented accommodation | 65 (47 to 79) | 71 (59 to 82) | 76 (68 to 86) | 72 (64 to 82) |
| % belonging to Baganda Tribe | 72 (64 to 77) | 65 (35 to 80) | 66 (57 to 71) | 62 (38 to 78) |
| % belonging to main religions |  |  |  |  |
| Catholic | 36 (29 to 40) | 36 (30 to 43) | 37 (34 to 38) | 36 (31 to 40) |
| Muslim | 25 (21 to 31) | 26 (21 to 32) | 24 (19 to 29) | 22 (16 to 29) |
| Protestant | 23 (17 to 31) | 24 (22 to 25) | 25 (18 to 29) | 26 (25 to 29) |
| Born Again | 13 (10 to 17) | 10 (9 to 13) | 13 (10 to 16) | 13 (10 to 19) |

^a^Unweighted mean of site-level summary data.

^b^ At follow-up, median number of households per selected EA=170 (interquartile range 125-250)

**Appendix 3b.** Characteristics of respondents to baseline and follow-up surveys

|  | Baseline | | | | Follow-up | | | |
| --- | --- | --- | --- | --- | --- | --- | --- | --- |
| Individual-level | Men number (%) | | Women number (%) | | Men number (%) | | Women number (%) | |
|  | Intervention | Control | Intervention | Control | Intervention | Control | Intervention | Control |
| Age (years) - mean(sd) | 27.1 (6.8) | 27.6 (7.0) | 28.4 (7.7) | 28.2 (7.7) | 28.6 (7.8) | 29.9 (8.2) | 28.4 (7.4) | 29.1 (8.2) |
| Above primary education | 275/419 (66%) | 321/447 (72%) | 157/374 (42%) | 140/343 (41%) | 556/768 (72%) | 457/634 (72%) | 394/599 (66%) | 343/529 (65%) |
| Does not earn money | 87/419 (21%) | 94/447 (21%) | 180/374 (48%) | 166/343 (48%) | 108/768 (14%) | 63/634 (10%) | 219/599 (37%) | 177/529 (33%) |
| Ever had a regular partner | 326/418 (78%) | 352/447 (79%) | 350/374 (94%) | 316/342 (92%) | 584/768 (76%) | 481/634 (76%) | 558/599 (93%) | 487/529 (92%) |
|  |  |  |  |  | Including casual: 689/768 (90%) | Including casual: 573/634 (90%) | Including casual: 574/599 (96%) | Including casual: 497/529 (94%) |
| Had a regular partner in past 12 months | 313/419 (75%) | 335/447 (75%) | 305/374 (82%) | 274/343 (80%) | 545/768 (71%) | 435/634 (69%) | 486/599 (81%) | 401/529 (76%) |
|  |  |  |  |  | Including casual: 624/768 (81%) | Including casual: 525/634 (83%) | Including casual: 504/599 (84%) | Including casual: 427/5292 (81%) |
| Currently married/cohabiting | 165/419 (39%) | 191/447 (43%) | 228/374 (61%) | 205/343 (60%) | 407/768 (53%) | 314/634 (50%) | 377/599 (63%) | 286/529 (54%) |
| In polygamous marriage (among those married) | 37/165 (22%) | 45/191 (24%) | 49/201 (24%) | 57/187 (30%) | 36/407 (9%) | 38/314 (12%) | 53/316 (17%) | 57/246 (23%) |
| No children | 237/419 (57%) | 223/447 (50%) | 83/374 (22%) | 83/343 (24%) | 351/768 (46%) | 267/634 (42%) | 136/599 (23%) | 121/528 (23%) |
